# Optimizing patient-centeredness in the transitions of healthcare systems in low- and middle-income countries

**DOI:** 10.1186/1472-6963-14-386

**Published:** 2014-09-12

**Authors:** Yodi Mahendradhata, Aurélia Souares, Revati Phalkey, Rainer Sauerborn

**Affiliations:** Center for Health Policy and Management, Faculty of Medicine, Gadjah Mada University, Sekip Utara, Yogyakarta, 55281 Indonesia; Institute of Public Health, Faculty of Medicine, University of Heidelberg, Heidelberg, Germany

**Keywords:** Patient centered care, Health services, Global health

## Abstract

**Background:**

Patient-centeredness is necessary for quality of care. Wide-spread incorporation of patient-centered practices across the health system is challenging in low and middle income countries (LMICs) given the complexity of scarce resources, competing priorities and rapidly changing social, economic and political landscapes. Health service managers and policy makers in these settings would benefit from a framework that allows comprehension and anticipation of forthcoming challenges for optimizing patient-centeredness in healthcare delivery. We set out to formulate such a framework, based primarily on analysis of general patterns of healthcare system evolution in LMICs and the current literature.

**Discussion:**

We suggest that optimization of patient-centeredness in LMICs can be thought of as occurring in four phases, in accordance to particular patterns of macro transitions. Phase I is characterized by a deeply fragmented system based on conventional clinical approaches, dealing primarily with simple acute conditions. In phase II, the healthcare systems deal with increasing chronic cases and require redesign of existing acute-oriented services. In phase III, health services are increasingly confronted with multimorbid patients, requiring more coordinated and integrated care. Complex health care needs in individual patients are increasingly the norm in Phase IV, requiring the most optimal form of patient-centered care. This framework helps to identify and map the key challenges and implications for research, policy and practice, associated with the transitions ahead of time.

**Summary:**

We have developed a framework based on observed patterns of healthcare and related macro-transitions in LMICs. The framework provides insights into critical issues to be considered by health service managers and policy makers.

## Background

Patient-centered care is a universal necessity. The Institute of Medicine (IoM) - the health arm of the United States National Academy of Sciences - considers it one of the key elements of high quality care and describes it as “providing care that is respectful of and responsive to individual patient preferences, needs, and values, and ensures that patient values guide all clinical decisions [1]”. IoM has further elaborated the dimensions of patient-centered care as: (1) respect for patients’ values, preferences and needs; (2) coordination and integration of care; (3) information, communication, and education; (4) physical comfort; (5) emotional support; and (6) involvement of family and friends. Patient centeredness has been shown to lead to better patient satisfaction; outcomes; quality of life and improved care utilization [2–5].

The care that patients in low- and middle-income countries (LMICs) have been receiving has undoubtedly been vastly improved, however, much is left to be achieved [6–8]. Consider the following case study, which illustrates the lack of patient-centered care in a middle income country: “Mr X is a 60 years old patient diagnosed with Diabetes Mellitus (DM) and hypertension eight years ago. His internist prescribed him a regimen consisting of seven drugs, which he has adhered to diligently, as well as coming in for monthly check-ups at a hospital. Two-months ago he started to suffer from persistent cough. He went to a pulmonologist at another hospital, who diagnosed him with Tuberculosis (TB) and additionally prescribed a treatment regimen consisting of five drugs. During the course of treatment he experienced nausea, vomiting and skin rash. He seriously considered discontinuing the drugs. He then consulted both doctors separately, who apparently had conflicting opinions and had not communicated with each other. Thus, he is left to make his own decision on the continuation of his treatment, based on conflicting advices”.

Uncoordinated care, as illustrated above, is one of the major issues that limits effective patient management [9]. Other major issues include: poor communication and provision of information; poor organisation of service delivery and long waiting times; insufficient facilitation of self care; and lack of patient and carer involvement in decision making. Patients are dissatisfied with the quality of the interaction with their provider, as many providers focus on the disease alone rather than on the patient [10].

Similar cases are also found in high-income countries, however, the challenges are evidently much greater in LMICs as policymakers struggle with scarce resources, competing priorities and rapidly changing environments. These challenges notwithstanding, patient-centeredness is feasible even in LMICs [11]. The challenge is how to realize it sustainably and on a large scale, given the complex environment. Thus, we set out to formulate a framework for policymakers and health service managers to aid in their comprehension and anticipation of challenges, for optimizing patient-centered care in these countries. We base it on an in depth analysis of general patterns of healthcare systems in LMICs and current literature.

### A framework of healthcare system transitions in LMICs

Societies have a life cycle [12]. Omran depicted how disease patterns evolve over time in societies, in response to, among other factors, demographic transition and economic development, resulting in an epidemiological transition [13]. Accordingly, infectious diseases and nutritional deficiencies are predominate in a society in which the majority of the population is young. Although chronic noncommunicable diseases (NCDs) become more prevalent as the population becomes more mature, infectious diseases still prevail. NCDs predominate in an aging population. Rayner and Lang [14] more recently proposed a longer list of macro-transitions to establish a framework for ecological public health. Some of these transitions are relatively familiar to the global health community (e.g. urban, nutrition, biological), others are less familiar (e.g. cultural, democratic, energy). They assert that all of those transitions shape health and that none of the transitions should be viewed in isolation as it is their totality that is significant. This inevitably leads to complexity, but need not paralyse public health thinking and action [14].

The complex patterns of macro-transitions arguably have consequences for healthcare systems, including their extent of patient-centeredness. Notably, high-income countries experienced population ageing after they became wealthy; most LMICs, however, will have to cope with such transitions prior to becoming wealthy. This brings us to the critical question of how can LMICs be offered a lead time in facing the inevitable transitions. A pragmatic way forward is, thus, to conceptualize how healthcare systems should be systematically transformed in response to these multiple transitions in LMICs.

**Figure 1:**
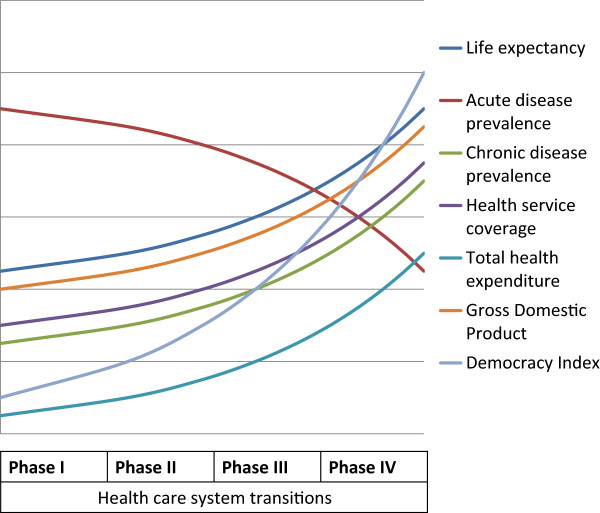
**Illustration of potential health care system transition pattern in response to multiple macro-transitions in low- and middle-income countries.**

We suggest that healthcare systems in LMICs can transform over time, towards an optimal form of patient-centeredness, both as a result and as a response to large scale transitions. Such transformation can be thought of as occurring in four generally sequential phases along a continuum: from a deeply fragmented system dealing primarily with simple conditions based on conventional clinical approaches to an integrated system equipped to deal with complex conditions based on interdisciplinary approaches. These phases are both driven by and are in response to macro transitions that evolve simultaneously. The patterns of interactions between the phases and the transitions would vary, but certain typical patterns should be identifiable and investigated through further studies. We present an illustration of a potential pattern (Figure 1). The phases are elaborated below. Table 1 presents a summary of the phases while Table 2 presents illustrative action points.

**Table 1:** **Phases of healthcare system transitions in low- and middle-income countries**

	Typical patient profile	Example of a typical case	Typical healthcare system feature
Phase I	Patients with acute conditions	Children with malaria	Acute care
Phase II	Patients with chronic condition	Elderly patients with lung cancer	Chronic care in parallel with acute care
Phase III	Patients with comorbidities	Patients with Tuberculosis and Diabetes	Integrated care
Phase IV	Complex patients	Single-parent with two children, Diabetes, obese, smoking, alcoholic, recently unemployed	Individualized/customized care

**Table 2:** **Illustrative action points for key stakeholders in different phases of healthcare system transitions**

	Phase I	Phase II	Phase III	Phase IV
Health service manager	Promote healthcare that is respectful of patients and families	Redesign services to meet requirements of acute and chronic conditions	Strengthen coordination of care; promote interdisciplinary teamwork for managing multi-morbidities	Promote institutional policies that minimize disruptions of effective communications and foster customized/ individualized care
Policy maker	Formulate policies to systematically integrate vertical programs into general healthcare services	Establishing task forces/working groups at national level to align interventions for acute and chronic conditions simultaneously	Establish policies and programs to support generalists and inter-professional education for managing multi-morbidities	Setting targets that will allow healthcare leaders to measure progress toward customized/individualized care
Donor	Provide more funding on and technical assistance for strengthening general healthcare services	Provide funding and technical assistance for designing strategies and services to address acute and chronic conditions simultaneously	Provide technical assistance for strengthening integrated primary care for multi-morbidities	Provide platform for sharing best practices in customized/individualized care
Scientific community	Provide evidence for shifting toward more horizontal health system; Develop integrated clinical diagnosis and treatment algorithm	Investigate potential synergies in policies, programs and services for addressing acute and chronic conditions	Studies to develop guidelines for care of patients with multiple conditions; models of coordinated care; shared decision-making and strategies to deal with conflicting priorities	Studies to support continuous enhancement of efficient flexible care management system that can respond to patients’ needs for different levels of support

### Phase I

In this initial stage, there is very limited recognition of the need for patient-centered care. The typical patients are young with acute (mainly communicable) conditions. This phase is characterised by a fragmented health system, dominated by multiple vertical programmes. These programmes are centrally administered, heavily controlled and focus on a few specific conditions (commonly linked to current donors’ priorities, e.g. Malaria) to maximize likelihood of impact [15]. Healthcare services in these settings are designed to manage episodic visits of acute conditions. Healthcare workers are limited, scarce at the periphery, and trained primarily to diagnose and treat acute conditions. They routinely diagnose and treat as many patients as possible within the limited resources, thus optimizing their capacities, even in routine care.

Many of these countries are entering the epidemiologic transition and would benefit from early preparedness, through studies documenting disease trends and studies on optimizing care for chronic communicable diseases, e.g. HIV/AIDS. They also need evidence for shifting to a more horizontal system, integrating specific disease control interventions into general healthcare services. In this context, they would benefit from development of integrated clinical diagnosis and treatment algorithm, Strengthening general healthcare system should be a priority for policy makers in this phase. In the meanwhile, health service managers should ensure that care is provided in a manner that is respectful to patients and their families.

### Phase II

The second phase starts with the recognition of an increase in patients with non- communicable diseases. The socio-economic conditions are gradually improving and life expectancy rises. Treatment adherence becomes increasingly important as more patients need drugs for a longer period. Patients are more knowledgeable of their diseases and demand better care. There is growing awareness of the inadequacy of health services (originally designed to manage acute conditions), leading to efforts to adapt best practices of chronic care from developed countries and from locally existing chronic communicable disease services, e.g. Tuberculosis (TB) [16].

These countries are experiencing a dual burden of communicable and non-communicable diseases [17]. There are shared features across these disease categories, such as common risk populations and the need for treatment adherence. Thus, there is arguably an opportunity to harness a combined approach. Unfortunately, the experts, institutions and policies that support management of communicable and non-communicable diseases in these settings, commonly remain as separate entities with limited interaction and alignment. There is an imminent risk of competing for funds to control either problem, rather than a fight against the double disease burden [12]. Hence, in order to move forward beyond this phase, there is a critical need to systematically document co-morbidities, particularly of communicable and non-communicable diseases, and identify and target potential synergies in case management. This needs to be complemented at the policy level by establishment of task forces/working groups aligning interventions to address both disease categories, capitalizing on existing capabilities, without competing for resources [16]. In the meanwhile, health service managers need to ensure that care is capable of effectively meeting the needs of acute and chronic conditions simultaneously.

### Phase III

In this phase, there is rising awareness that many patients are presenting with more than one disease condition. The HIV epidemic demonstrated that the risk for developing TB can increase over four fold where both diseases are prevalent [12]. There is an increase in the simultaneous incidence of communicable and non communicable diseases in many patients. The interaction of diabetes with TB was first recognized several years ago, but subsequently forgotten by clinicians and public health experts, until diabetes rose exponentially in TB-prevalent LMICs [12].

People with multimorbidity, in general, have a worse quality of life [18, 19]. Expenditure on health care rises almost exponentially with the number of disorders that an individual has, and therefore, increasing multimorbidity generates financial pressure. The economic burden heightens the need to manage people with several illnesses in an efficient way. Furthermore, healthcare providers in LMICs generally see patients during brief visits. When patients have multiple conditions, screening, counseling, and treatment needs exceed the time available [20]. Thus, the healthcare providers are unable to meet these multiple demands, further complicated by inadequate system support and little guidance about how to manage multimorbid patients. These countries are eventually faced with a fundamental challenge to the single-disease focus that pervades conventional medical care.

Fundamentally, people with multimorbidity need more coordinated and diverse care [21]. Use of poorly coordinated services to manage individual diseases is inefficient, burdensome and unsafe [18]. Policy makers and health service managers, thus, need to ensure that approaches focusing on single diseases are complemented by support for generalists (mainly in primary care) providing continuity and coordination for people with multimorbidities. There is, therefore, a need for studies about strengthening integrated primary care, including studies that contribute toward: guidelines for caring for patients with multiple conditions; models of coordinated care; shared decision-making and strategies to deal effectively with conflicting priorities [22].

Socioeconomic, behavioral and environmental circumstances can also affect health outcomes and contribute to complexity [23]. Care plans need to be adjusted to address these issues, as they can become barriers to reaching congruence between patient and provider, leading to low treatment compliance and diminishing the physician’s effectiveness in optimizing patient’s health. Hence, there is a need to inform the care of complex patients, through interdisciplinary research, to lay the foundation for in-depth understanding of the multiple sources of patient complexity (e.g. biological, socioeconomic, behavioral, environmental) and to better understand how care provision should be harmonized to optimize patients’ health. Thus, health service researchers need to work with physicians, social scientists, complexity theorists, network experts, education experts, behavior analysts and scientists from other relevant disciplines to carry out much needed interdisciplinary studies on patient complexity in healthcare.

### Phase IV

In the final phase, complex patients are increasingly the norm, rather than the exception. The features of the conditions range from complex to chaotic (unstructured randomness) [24]. There is a broad consensus among stakeholders, in this phase, that the purely biomedical model is inadequate and that patient management needs to be grounded in an alternative model, in which illnesses result from complex interactions between different system components [25].

In this phase, policy makers commit themselves to a coherent and coordinated effort to advance patient-centered care, setting targets that will allow healthcare leaders to measure progress toward patient-centeredness [26]. There is a clear and focused national policy to promote patient-centered care. Such policy drives healthcare organizations to move towards a culture of patient-centeredness by supporting patients in shared decision making and self- management. Such policies also encourage healthcare workers to acquire and maintain interpersonal competencies through intensive continuous education. Health service managers promote a culture of patient-centeredness through policies that minimize disruptions in effective communications and foster therapeutic relationships.

As the population of complex patients grows, there is an increased demand for more intensive care management that may drain already limited resources [27]. Thus, research in this phase is needed to continuously tweak the system to more efficiently deliver optimum patient-centered care, including developing efficient flexible care management systems that can respond to patients’ needs for different levels of support in a timely manner [27], i.e. customized/individualized care.

## Discussion

We have presented a framework of healthcare system transitions in LMICs toward an optimum level of patient-centeredness. We are not the first in suggesting that macro transitions define the terrain within which health systems operate [12–14]. Our focus, however, represents a step forward in conceptualizing how LMICs could anticipate, prepare, and respond pragmatically to dynamic large scale transitions.

Needfully, the framework is a simplification of the complex reality: some phases may not be sequential as healthcare systems are constantly evolving and complex. Our framework is not meant to be prescriptive or predictive. These systems are non-predictable, but potentially comprehensible by observation and pattern recognition. The framework describes these observed patterns, examining the process of overall change and attempts to break it into manageable parts for policymakers in LMICs. It seems to provide insights into pragmatic steps which could be taken prior to subsequent macro-transition waves.

From a theoretical construction perspective, this framework is an initial proposition. It would evidently still need to undergo subsequent empirical testing, refinement, affirmation and extension. In the meanwhile, however, we believe that sharing the current proposition can promote deeper understanding of healthcare system transitions and optimization of patient-centeredness by stimulating investigative studies and discussions.

Dialogues on healthcare system transitions and optimization of patient-centeredness are now timely, as many LMICs are gearing up for universal coverage, with increasing focus on requirements for effective coverage [28]. Patient-centeredness is clearly required for effective care [29]. A unique window of opportunity is currently available to progress toward patient-centered care in LMICs. We recommend that the systematic transition for optimizing patient-centeredness now becomes a focal element for universal coverage policy in LMICs.

## Summary

Patient-centeredness is a necessity for quality care, including care in low- and middle-income countries (LMICs). We present a conceptual framework for health service managers and policymakers in LMICs to better comprehend the challenges for optimizing patient-centeredness, through ongoing multiple macro-transitions. The framework describes observed general patterns, examines the processes of overall change and breaks it into smaller more comprehensible and, hence, manageable parts. These parts have been laid out in a continuum with four distinct phases, starting from a deeply fragmented system dealing primarily with simple conditions based on conventional medical approaches, to a highly integrated system equipped to deliver customized care for patients with complex conditions. The framework is not meant to be prescriptive nor predictive, rather it is meant to promote a deeper understanding of healthcare system transitions for optimizing patient-centeredness, by stimulating further investigations and discussions. Such discussions are timely as health systems in LMICs are currently gearing up for universal coverage, with increasing emphasis on requirements for effective coverage.
